# Coherence Potentials Encode Simple Human Sensorimotor Behavior

**DOI:** 10.1371/journal.pone.0030514

**Published:** 2012-02-03

**Authors:** Dhanya Parameshwaran, Nathan E. Crone, Tara C. Thiagarajan

**Affiliations:** 1 National Centre for Biological Sciences, TIFR, Bangalore, India; 2 Department of Neurology, The Johns Hopkins University School of Medicine, Baltimore, Maryland, United States of America; Claremont Colleges, United States of America

## Abstract

Recent work has shown that large amplitude negative periods in the local field potential (nLFPs) are able to spread in saltatory manner across large distances in the cortex without distortion in their temporal structure forming ‘coherence potentials’. Here we analysed subdural electrocorticographic (ECoG) signals recorded at 59 sites in the sensorimotor cortex in the left hemisphere of a human subject performing a simple visuomotor task (fist clenching and foot dorsiflexion) to understand how coherence potentials arising in the recordings relate to sensorimotor behavior. In all behaviors we found a particular coherence potential (i.e. a cascade of a particular nLFP wave pattern) arose consistently across all trials with temporal specificity. During contrateral fist clenching, but not the foot dorsiflexion or ipsilateral fist clenching, the coherence potential most frequently originated in the hand representation area in the somatosensory cortex during the anticipation and planning periods of the trial, moving to other regions during the actual motor behavior. While these ‘expert’ sites participated more consistently, other sites participated only a small fraction of the time. Furthermore, the timing of the coherence potential at the hand representation area after onset of the cue predicted the timing of motor behavior. We present the hypothesis that coherence potentials encode information relevant for behavior and are generated by the ‘expert’ sites that subsequently broadcast to other sites as a means of ‘sharing knowledge’.

## Introduction

Theory suggests that activity propagating among subsets of neurons in the cortex create transient structures or ‘engrams’ that drive thought and behavior. These transient structures might be the physiological equivalent of a percept or an integrated view of the organism with respect to the external world [1], [2], [3], [4], [5]. However, the nature of the relationship between propagating activity and behavior is unclear.

A number of seemingly disparate pieces of the puzzle have been discovered. Stimulation of specific sites in the brain leads to movement in particular body parts or specific sensations suggesting that function is highly localized [6], [7]. Conversely, individual neurons have been found to encode information about specific stimuli and features in the external world, showing a high degree of localization by sensory modality [8], [9], [10]. However, studies of individual neurons in both rats and monkeys show that responses to stimuli are highly variable and neurons rarely respond to the same stimulus in 100% of the trials [11], [12], [13], [14]. Rather the response variance of individual neurons is typically more than 100% of the mean response [15], [16]. Further, while neighbouring neurons are often similar in their response profile, there are many cases where they are not [17], [18], [19]. Complicating matters further is that cases of cross modal responses have been found. For instance 16% of ostensibly ‘unimodal’ neurons in the visual cortex respond to both visual and auditory stimuli [20], [21], [22], [23], [24]. Thus while there is a gross level of localization, there is still high variability and heterogeneity. The heterogeneity is even greater when it comes to more downstream responses such as decision tasks [25]. Indeed early studies by Lashley and Hebb indicate that information about the external world is distributed across the cortex and learned behaviours are not easily destroyed by localized lesions [3], [26].

Somehow multi-sensory information must come together to create an integrated view in a manner that reconciles both functional localization and distributed memory. It is clear that there is a high degree of lateral connectivity in the cortex as well as long range propagation across functional regions [27], [28]. Furthermore, both local and long range synchrony in the both spiking and local field potential activity have been found to be associated with both stimulus presentation and behaviours suggesting that amplitude and spectral synchrony may be key indicators of propagation that forms a meaningful assembly of activity [1], [6], [29], [30], [31], [32], [33]. Notably, stimulus presentation also results in widespread synchronization of membrane potentials at high frequencies even among neurons with different stimulus response profiles [34]. Typically, however, such synchrony can be studied only as a statistical phenomenon over multiple trials leading to difficulty in identifying synchrony at particular instances.

More recently, a new phenomenon has been reported called Coherence Potentials whereby when activity reaches a high level of synchrony in the local field it can spread rapidly within the cortex in cascading, saltatory fashion without any distortion of its temporal structure [35]. Coherence Potentials might thus be thought of as network level action potentials with waveforms that are typically a few hundred milliseconds in duration. However, because each Coherence Potential cascade is highly distinct in its temporal structure rather than a stereotypical waveform, it has many degrees of freedom to encode different information. This suggests that coherence potential cascades may represent the spread of information, recruiting sites across the cortex into a transient ‘agreement’. Significantly, the matching of a relatively unique temporal structure of distant sites also allows for more reliable identification of individual instantiations of synchrony or agreement.

Thus far, however, the phenomenon of coherence potentials has been demonstrated only in the spontaneous activity of monkeys [35]. It is therefore unclear if this phenomenon is identifiable in humans and how it relates to behavior. Here we demonstrate the phenomenon in human ECoG during a behavioral task and describe a clear relationship to a simple motor behavior. The relationship is not as expected and suggests new hypothesis that can reconcile functional localization and distributed memory.

## Results

### Visuomotor task and functional mapping

**Figure 1:**
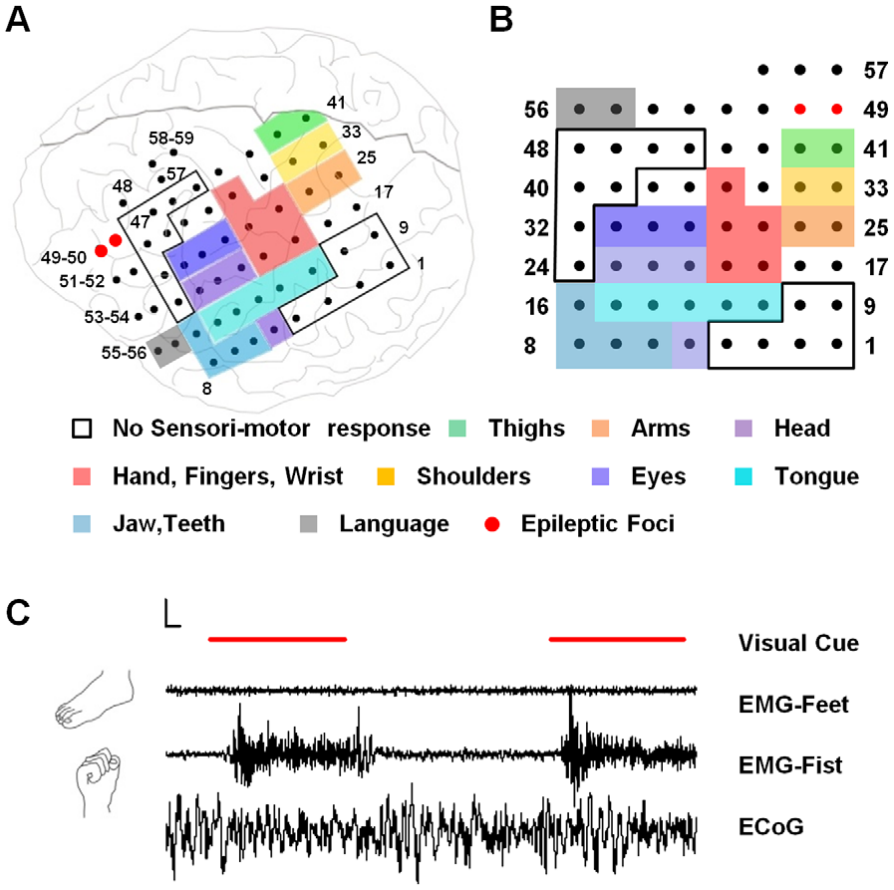
Functional mapping and experimental setup. (**A**) Functional map created by stimulation of electrode pairs in a subdural electrode array (black dots) implanted in the left fronto-parietal cortical region in a human subject. The colors represent distinct sensorimotor response profiles of movement and/or sensation in the arm, hand and face as well as non-sensorimotor responses such as deficits in language comprehension. The numbers indicate the electrode #. Electrodes 49 and 50 produced seizures on stimulation. (**B**) Visualization of the electrode array (shown in Figure 1A) transformed on to a symmetric 8×8 grid used in subsequent figures. (**C**) The subject was asked to mimic the visual cue (red line; either right or left fist clenching or right or left foot dorsiflexion) for the duration it was shown on a screen. Each visual cue was presented 50 times for 3 s followed by an interval varied randomly between3 and 5 ms. Motor responses were monitored simultaneously with surface EMG from both the wrist and foot muscles for all 4 tasks. ECoG traces (bottom) were simultaneously recorded from all 59 electrodes. Scale bar – 300 mV, 300 ms.

Here we analyzed the relationship between coherence potentials and a simple motor behavior using electrocorticograph (ECoG) recordings from a 31 year old male implanted with 59 subdural electrodes in the fronto-parietal region of the left hemisphere (Figure 1A, shown transformed onto a symmetric grid in Figure 1B). Electrical activity was recorded simultaneously at each of the 59 electrodes over the course of four simple visuomotor tasks along with electromyography (EMG) responses from both hands, both feet and tongue. Each task involved imitating a motor behavior (right fist clenching, left fist clenching, right foot dorsiflexion and left foot dorsiflexion) that appeared on a screen until it went off. Each cue was presented 50 times in repetitive succession with a variable 3–5 second pause between trials. Figure 1C shows the cue presentation along with the EMG recording in the right hand and right foot and the ECoG recording at one electrode over two trials of the fist clenching task.

Since this electrode implantation was carried out in a patient about to undergo surgical resection for treatment of epilepsy, experiments were carried out to map the seizure foci and function by cortico-electrical stimulation between pairs of electrodes [6], [7], [30]. This functional map is represented in Figures 1A and 1B as a color code and provides insight into which regions of the cortex would be expected to be involved with carrying out the tasks of fist clenching and foot dorsiflexion outlined above. Five electrodes (# 19–20, 27–28, 36, highlighted in red) responded to stimulation with sensation and movement in the right (contralateral) hand, fingers or wrist (Hand region). Among these, electrodes 27 and 28 were highly specific to the digits (fingers excluding thumb) which are the first part to move in a fist clenching behavior. In contrast, there was no response of the foot or leg other than the right thigh (# 41–42). However there was significant coverage above the neck (various shades of blue) including head (electrode # 7, 21–23), tongue (electrode # 11–15), jaw-teeth (electrode # 6–8, 16) and eyes (electrode # 29–31) as well as regions such as shoulder and arms. Two electrodes (#49, 50 shown in red) initiated seizure activity but were not close to the hand area. In addition, stimulation of several electrodes outside the sensorimotor region resulted in impairment in language comprehension (#51–56) or did not result in any detectable response (# 49–50, #57–59).

### Coherence Potentials in Human ECoG

Coherence potentials are large amplitude periods in the local field potential lasting up to a few hundred milliseconds, that spread across the cortex in saltatory fashion on millisecond time scales without any distortion of their waveform or amplitude [35]. These are complex waveforms that differ in each instance and appear to reflect the temporal pattern of underlying neuronal spiking behavior [35], [36], [37]. Consequently they could potentially carry highly complex and varied information across the cortex without distortion. Sites participating together in a coherence potential can therefore be identified by extracting the periods of negative excursions of the LFP (nLFPs) at each site and comparing their temporal structure using a simple correlation of their time series.

**Figure 2:**
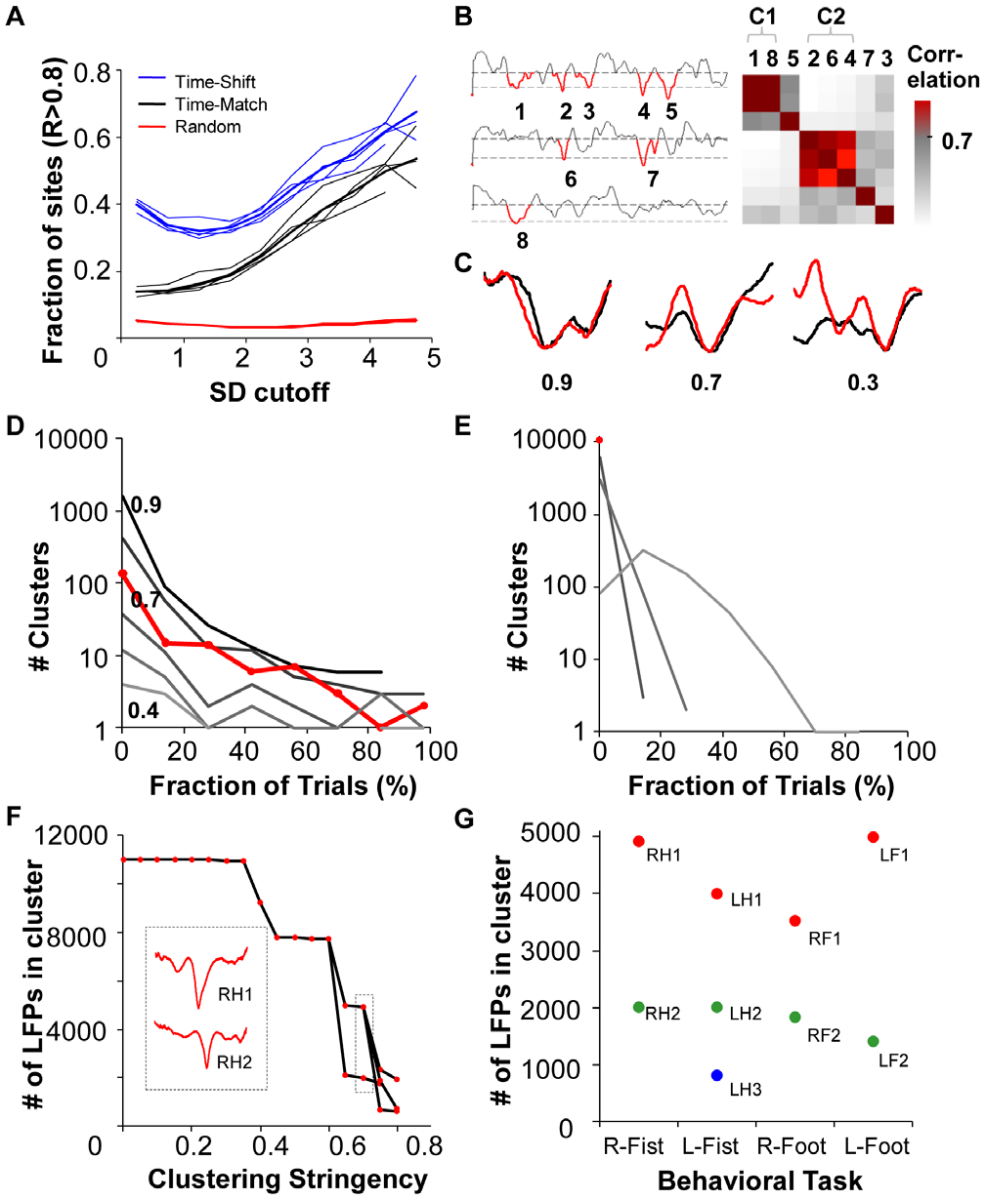
Trial Associated Coherence Potentials. (**A**) Fraction of sites with identical waveform (correlation R≥0.8) arising simultaneously (Time-Matched) or within ±10 ms milliseconds (Time-Shifted) increased non-linearly with increasing nLFP amplitude. Time-matched and time-shifted correlations calculated at 300 randomly selected sites per electrode could be fit to a sigmoidal function with an R^2^ of 0.99 and 0.95 respectively. (Light colors: values for each of the four behavioral tasks, dark lines: mean). Comparisons between randomly selected sites shown in red. (**B**) Large amplitude nLFPs (peak<-2SD from the mean; numbered 1–8 for 3 electrodes shown) were identified at all 59 electrodes across the entire recording spanning all 50 trials. Correlations between all pairs of identified nLFPs were calculated after aligning their peaks. The nLFPs were then grouped using a clustering algorithm based on their correlation values (8×8 correlation matrix shown here). (**C**) Three pairs of nLFPs with correlation values of 0.9, 0.7 and 0.5 show that increasing correlation reflects increasing similarity of temporal structure. (**D**) Cluster dispersion across trials for different correlation criteria for clustering in the right fist clenching task. At a low correlation criterion (light gray) all nLFPs collapse into a few clusters and thus span all trials (trial-spanning clusters). At higher correlation stringency, clusters splintered and only a few clusters persisted across all trials. R = 0.7 was chosen for further analysis. (**E**) Amplitude shuffled ECoG traces did not produce clusters that spanned all 50 trials. At high clustering stringencies (0.7) all clusters had only 1 nLFP demonstrating that these cascading nLFPs are a non-random phenomenon. (**F**) Splintering of trial-spanning clusters with increasing correlation criteria for clustering (right fist clenching task shown here, other tasks shown in Figure S1). The lines connect the parent cluster from which smaller clusters have separated. Only trial-spanning clusters are shown in the figure. Two clusters persist across all trials even for high correlation criterion. Inset: Mean traces of the nLFPs in the two clusters at R = 0.7 are shown in the same order. (**G**) Cluster sizes (number of nLFPs) of the nine trial-spanning clusters from all four behavioral tasks. (Colors and the cluster names shown are used in subsequent figures).

In multi-electrode array recordings from rat and monkeys, as the amplitude of the nLFPs increased, the probability of finding mirroring sites within a few milliseconds increased according to a sigmoidal function – the hallmark of the nonlinear nature that defines this phenomenon [35], [38]. We found similar phenomenology in these human ECoG recordings, although the spatial resolution of the recording was substantially lower (electrodes were 4 mm in diameter compared to 30 µm in earlier studies). As the nLFP amplitude increased, the sites with identical waveform patterns arising within millisecond timescales increased nonlinearly (Figure 2A) and could be fit with a sigmoidal function with an R^2^ of 0.99 and 0.95 with the time-matched and time-shifted correlations respectively. The sigmoid shape indicates a threshold-like non-linear transition to high spatial coherence as a function of amplitude. In contrast, comparisons of random periods of increasing amplitude remained constant. This establishes the presence of coherence potentials in humans across much larger spatial areas than seen before (over ∼44 cm^2^ of cortex compared to 64 mm^2^ shown previously in monkeys).

To identify Coherence Potentials that might recur or extend over longer time scales (i.e. the entire duration of the recording) we used a different time independent approach. We identified all the nLFP periods in all electrodes across all 50 trials for each task that met this criterion, and then compared their waveform similarity using a simple correlation measure. While the particular threshold that defines the coherence potential is ambiguous in the sigmoid, we chose the amplitude value at a midpoint of the rise phase (≤- 2SD of the electrode mean voltage) as a cut-off for analysis in relation to the behavioral trials. Correlation values comparing 8 nLFPs to one another (Figure 2B, right) are shown as a matrix that is ordered based on a clustering algorithm that groups the nLFPs based on a similarity criteria (i.e. a minimum mean all-to-all correlation value which we call the *correlation criteria for clustering*, see Methods). Figure 2C shows examples of correlation values for different pairs of waveforms. A cluster would be considered to be a single coherence potential cascade or repetitive cascades of the same coherence potential. The size of clusters in terms of the number of nLFPs distributed with a heavy tail that was power-law like and has similarity to the distribution of neuronal avalanches (Figure S3) which are nLFPs that are clustered based on temporal rather than waveform similarity criteria [36], [39].

### Trial Locked Coherence Potentials

We reasoned that if a coherence potential carried relevant information about the task then it should recur consistently in each trial, time locked in some manner to the task. We therefore looked for clusters of these nLFPs that spanned all of the 50 trials for each task (Figure 2D, right fist clenching, Figure S1, all other tasks). Because the trials were repetitive with intervals of only a few seconds, we considered the start of a new trial as the time when the EMG relaxed after the preceding trial, when the patient would begin anticipating the next trial. When the correlation criterion for clustering was low, all of the nLFPs collapsed into very few clusters that typically spanned most or all of the trials. With increasing clustering stringency, the clusters fragmented such that most clusters spanned very few trials. Nonetheless, even for high correlation criteria of 0.7 and 0.8 a small number of clusters persistently spanned 100% of trials. When the same analysis was carried out after shuffling the time series on each electrode, such trial-spanning clusters of large amplitude nLFPs never occurred even for very low similarity criteria (Figure 2E). These cascades of similar large amplitude nLFP waveforms are thus highly structured, non-random phenomenon.

We then looked specifically at how the trial-spanning clusters fragmented with increasingly stringent clustering criteria (Figures 2F and S1). Since all large nLFPs are negative excursions, they would tend to be positively correlated. Thus even as the clustering criteria was increased from 0.05 to 0.35 a single cluster persisted. Beyond 0.35 the cluster rapidly fragmented into either smaller clusters, many of which no longer spanned all trials (and therefore were no longer shown on this plot) or into a few clusters that still spanned all trials. Interestingly, in all cases, as the similarity was increased from 0.75 to 0.85, the clusters did not fragment or lose nLFPs significantly but rather maintained their integrity, indicating that most nLFPs in the clusters were correlated at levels beyond the cut-off. We thus chose a cut-off of 0.7 as representing a high degree of similarity that was used for subsequent analysis [38] (Figure 2C). As a further check of cluster quality at this cut-off we looked at the average distance of all points to one another (*a(i))* within a cluster and found that they were distributed narrowly and fairly similarly across all clusters (Table S1). We then calculated the silhouette coefficients (SC; see Methods) to look at how well separated these clusters were from other clusters and found that more than 82% of nLFPs have SC values >0 indicating that they were well separated (Table S2).

At this clustering criterion we found between 476 and 567 clusters in each behavioral dataset, but only 2 or 3 that spanned all 50 trials (i.e. trial-spanning coherence potentials; Figures 2F, 2G and S1). The trial-spanning clusters had anywhere from 817 to 4997 nLFPs suggesting coherence potential cascades of 16 to 100 nLFPs on average in each trial (Figure 2G). We thus looked in detail at how these nLFPs were distributed in time within and across trials to determine if they might be separate repeating coherence potentials with a clear association to the behavioral task.

**Figure 3:**
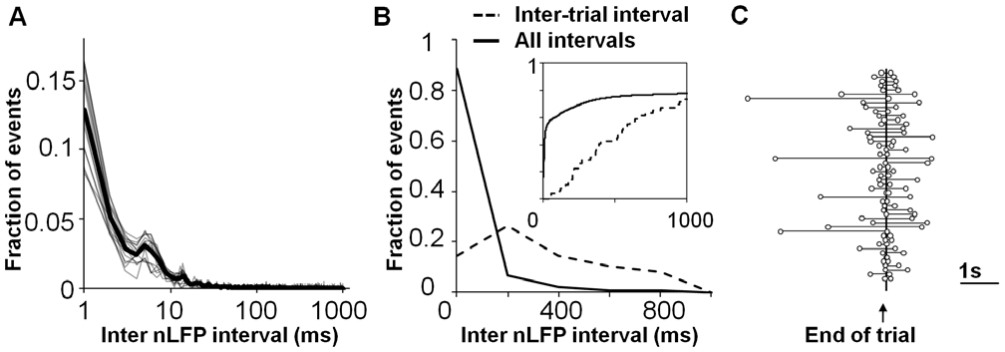
Coherence Potential Cascades Temporally Associated to Trials. (**A**) Histogram of the time difference between consecutive nLFPs (inter-nLFP interval) within each cluster (grey, thick black: average of all clusters) shows fast cascades with propagation times largely within 10 ms. (**B**) Distributions of the intervals between the last nLFP of one trial and the first nLFP of the next trial of a trial spanning cluster (RH1, dotted line) were significantly longer than the inter-nLFP intervals within the trials (solid line) indicating clusters are fast trial associated cascades punctuated by longer pauses at the end of the trial. (Inset: cumulative histogram) (Other tasks shown in Figure S2). (**C**) The pause between trials shown for all 50 trials for trial spanning cluster RH1. Each row represents a trial with the horizontal axis being time. The circles represent last nLFP before the end of the trial and the 1^st^ nLFP at the beginning of a new trial.

The distribution of inter nLFP intervals within each trial for each trial spanning cluster (Figures 3A and S2) followed a power-law like heavy tailed distribution where 66.39% of the intervals between nLFPs were <10 ms and many nLFPs occurred simultaneously (i.e. with an interval of 0) suggesting a fast one-to-many cascading propagation. In contrast, the intervals between the last nLFP of one trial (defined as the cessation of the motor behavior) and the first nLFP of the next trial (starting immediately after the cessation of the motor behavior) were orders of magnitude longer, between 39 ms and 4.2 seconds (Figures 3B, 3C and S2). The peak of the last nLFP typically occurred about 100 ms before the motor behavior ended (end of trial), a duration similar to the duration of the nLFP and the next nLFP occurred anywhere from 1.6 s to 4.9 seconds before the subsequent presentation of the cue. Thus the trial spanning clusters were composed of rapid Coherence Potential cascades that were punctuated by pauses at the end of the trial, clearly establishing a relationship to the trial and associated behavior.

### Spatiotemporal Organization of Trial-Associated Coherence Potentials

**Figure 4:**
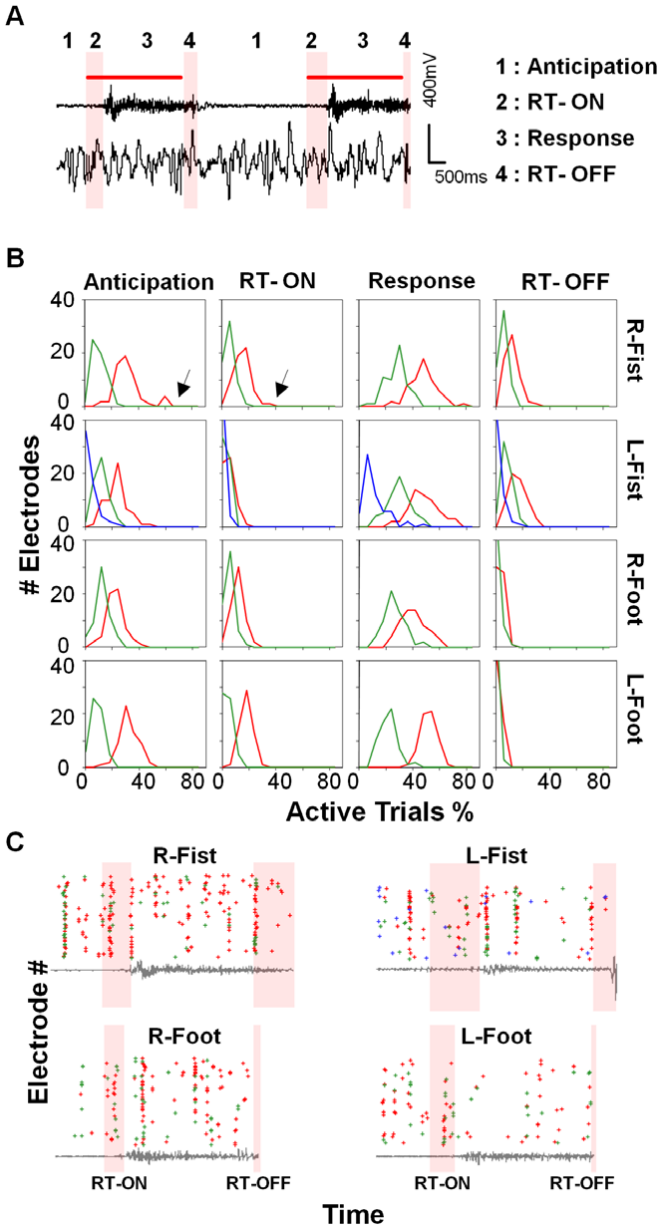
Trial-spanning clusters span all intervals. (**A**) EMG recordings (above) and corresponding ECoG recordings at one electrode (below) for 2 consecutive trials. Red bar indicates the visual cue for behavior. The EMG amplitude was used to define the start and stop of the motor response. Each trial was divided into 4 time intervals – 1, Anticipation (time prior to cue onset) 2, Reaction Time-ON (cue onset to initiation of motor behavior) 3, Response Time (duration of motor behavior) and 4, Reaction Time–OFF (cue off to cessation of motor behavior). Scale Bar Y, 200 mV X, 200 ms. RT-ON and RT-OFF shaded in pink. (**B**) Distribution of electrode participation across trials for each interval for each behavior (cluster color coding as in Figure 2G). In all clusters, each electrode participated only in a fraction of trials, typically less than 50% in each interval. A bimodal distribution in cluster RH1 (right fist clenching) indicates a small number of electrodes active in a much larger fraction of trials during the Anticipation and RT-ON intervals (marked by arrows). (**C**) Raster plot with electrodes 1–59 represented along the y-axis shows nLFP peak times in the trial-spanning clusters in a single trial (clusters color-coded as in Figure 2G). EMG traces for the trial shown in grey, RT-ON and RT-OFF shaded in pink.

We then analyzed the spatiotemporal organization of nLFPs within each trial in these trial-associated Coherence Potential cascades. To do so we separated each trial into four segments, Anticipation: the period from the end of EMG activity in the previous trial to the cue-ON of the next trial (taking only the last 1 second before the cue for this analysis, see Methods), Reaction Time –ON (RT-ON): the period from the onset of the cue to the onset of muscle movement (see Methods), Response Time: the duration of muscle movement, and Reaction Time – OFF (RT-OFF): the period from the time the cue went off to the ceasing of muscle movement (Figure 4A). The Anticipation period (time between trials) was randomly varied between 3 and 5 seconds. In the right fist clenching task, RT-ON was 410±152 ms (Mean ± SD) and RT-OFF 377±165 ms, while the motor response period was 2478±475 ms (see Table S3 for all tasks).

At one extreme we hypothesized that these cascades might be restricted in space and time to particular electrodes (for instance, just in the hand region) or particular intervals (for instance, just the reaction or response time), or at the other extreme, might be randomly organized in both space and time relative to the behavior.

At a gross level we found that in all clusters the majority of electrodes participated only in a fraction of trials in each of the four intervals (Figure 4B; 33±10% in Anticipation, 17±6% in RT-ON, 50±10% in Response and 13±5% in RT-OFF intervals for the R-Fist task. Also see, Table S4 for the other tasks) while only a few electrodes never participated. Moreover, in each cluster, the number of nLFPs in each interval varied widely between 0 and 200 across trials (Figure S4) indicating that the cascades were highly spatially heterogeneous. At first glance this would appear to suggest a random organization where there was neither spatial nor temporal specificity. However, in the clusters in the right fist clenching task, a small number of electrodes participated in a much larger number of trials in the anticipation and RT-ON period as indicated by a small secondary peak in the distribution (Figure 4B, marked by arrows) suggesting a scenario between the two extremes of stereotypical and completely random spatiotemporal cascades. Examples of these cascades for all clusters for each task are shown in Figure 4C.

Given that the functional mapping (Figures 1A and 1B) demonstrated that stimulation of particular electrodes were specifically responsible for movement of right digits (#27,28) or right wrist/hand (#19,20,36), we hypothesized that these electrodes would be more significantly engaged in driving the right fist clenching task but not others. Conversely, since no foot region was covered by the electrodes, we hypothesized that no electrodes would be clear drivers in these tasks.

**Figure 5:**
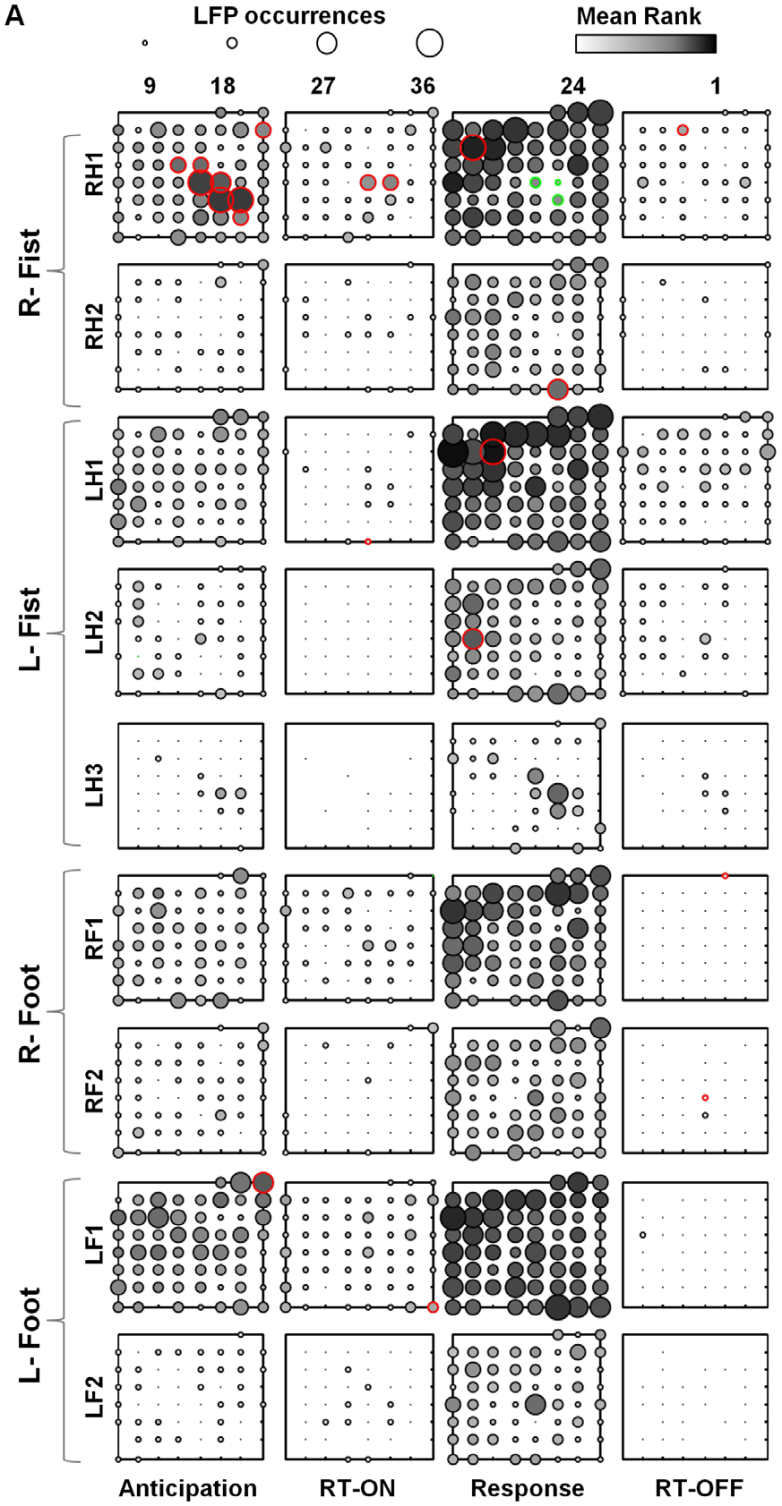
Spanning clusters show clear interval associated spatiotemporal patterns. (**A**) Spatiotemporal patterns of nLFPs in the 9 trial-spanning clusters for each interval in each of the four tasks. (Circle size: trial participation; Color intensity: mean rank of temporal occurrence early-black to late-white). Hand area electrodes have significantly increased activity (circled in red) in the Anticipation and RT-ON intervals that occurs early in time and significantly reduced activity (circled in green) in the Response interval.

To visualize spatial engagement patterns within each cluster we first plotted the percentage of trials in which each electrode participated as circles of varying size on a separate electrode grid for each interval (Figure 5A). To look at temporal engagement patterns we determined how often electrode sites that did participate occurred first (i.e. led the cascade) in any particular interval (not shown). In both cases we found that the same electrode sites were significantly prominent (Tables S5 and S6). We therefore created a composite spatiotemporal measure where we ranked electrodes according to the temporal order of nLFP occurrence in each interval for every trial and plotted these values on the electrode grid as color fills of different intensity (Figure 5A). If an nLFP arrived on two electrodes simultaneously, both electrodes received the same rank. If no nLFP occurred on the electrode during an interval in a particular trial, it was assigned a rank of 60. The mean rank over all 50 trials was then calculated. A rank of 1 for an electrode in a particular interval would thus indicate that the nLFP always occurred first at that electrode in every trial in that interval (shown as black) and a rank of 60 would mean it never participated. All ranks >25 are shown as white (Figure 5A and Table S7).

Indeed, the hand electrodes were particularly prominent in the anticipation and RT-ON periods of the right fist clenching (in cluster RH1) but not in any of the other tasks (i.e. ipsilateral fist clenching or ipsi/contralateral foot dorsiflexion). Specifically it was the digit electrodes (#27, 28) which temporally led the coherence potential cascade (RH1) after onset of the cue (i.e. in the RT-ON period). In the same cluster the digit electrodes were conspicuous by their lack of participation during the Response period. The hand region also appeared prominent in coherence potential LH3 in the ipsilateral (left) hand, although less so, during the motor response periods suggesting coordination between hand regions of both hemispheres. Interestingly, more frontal regions which were either associated with language impairment on stimulation or with no functional response had more prominent spatiotemporal participation during the motor response interval than any of the sensorimotor areas. Indeed when the RH1 cluster was ‘enriched’ by removing all nLFPs with silhouette coefficients <0.5, the significant hand and digit electrodes remained equally prominently in the anticipation and RT-ON periods indicating that they formed a tight core of the cluster (Methods, Figure S5).

One possibility was that the spatiotemporal prominence of the digit electrodes was simply due to a higher frequency of high amplitude nLFP events in the hand region that was not particularly related to the specific trials and intervals. To determine if this was the case we shuffled the timing of all the large nLFPs independently for each of the 9 trial-spanning clusters such that the coherence potentials in each of the 4 intervals retained the same number of nLFPs on each electrode but now in different time positions. We carried out this shuffling 5000 times calculating the probabilities of occurrence of the particular nLFP waveform in each interval for each electrode. The shuffled data showed an absence of prominence of particular electrodes in particular intervals. The probability that digit electrodes #27, 28 dominated anticipation and RT-ON with a mean rank <25 for the right fist clenching was <3 in 5000 shuffles (p<0.0005) and <3 in 5000 as percentage of trials in which they participated (p<0.0005). Conversely, in the right fist clenching clusters, the digit electrodes #27 and #28 were significantly less prominent than expected in the response interval. Electrodes with significantly higher rank than expected (p<0.005) are marked with red circles in Figure 5A while those with significantly lower rank than expected (p<0.005) are marked in green. Of note is that the distribution of p-values for all electrodes was bimodal with a small second peak arising at values <0.05 (Figure S6), a further indication of the distinction of specific sites as ‘experts’ or drivers of the particular cascade. Of note is also that the seizure sites (red, #49 and 50) participated below average in all intervals in all tasks.

### Coherence potentials on digit electrodes predict onset of fist clenching behavior

**Figure 6:**
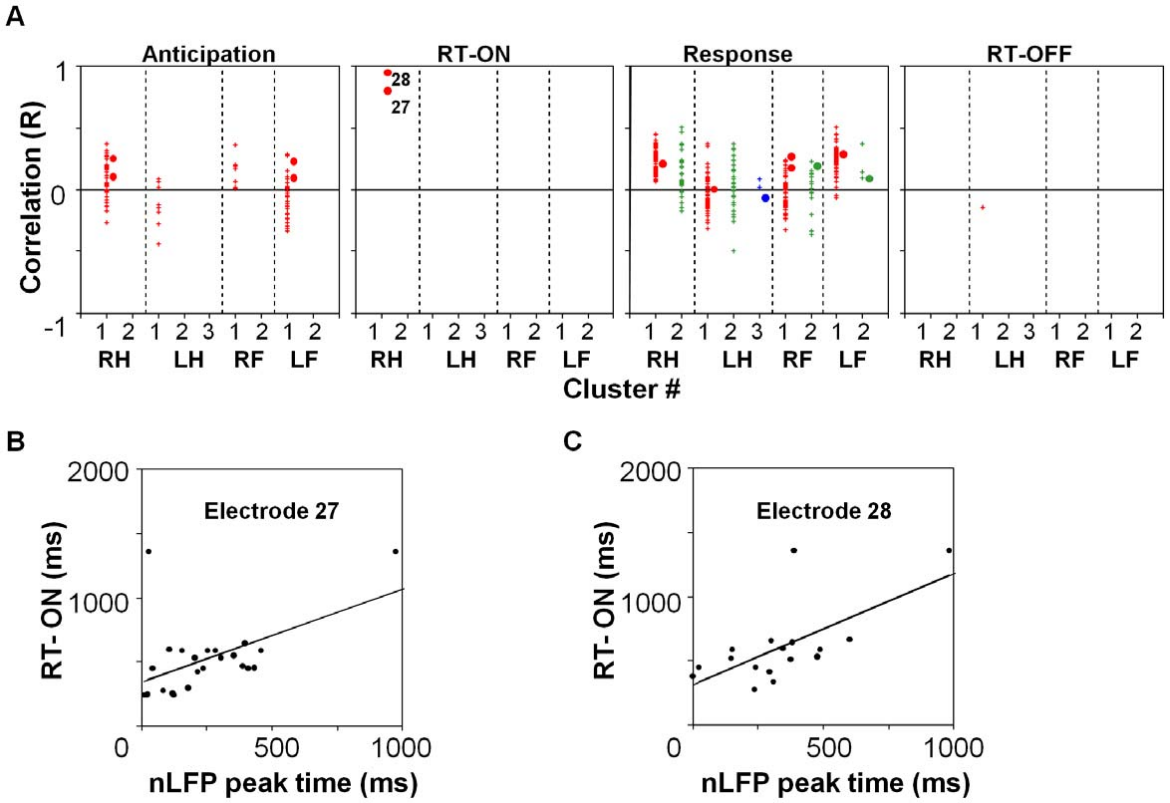
Coherence potentials predict response start. (**A**) Correlations between nLFP peak timing and motor behavior onset for the nine clusters in the four intervals show that timing on digit electrodes (#27 and #28) after onset of the cue (interval RT-ON) was highly correlated with the timing of muscle contraction. (Only electrodes participating in ≥30% of the trials were considered). (**B–C**) Timing of motor behavior plotted against nLFP peak timing for electrodes 27 and 28. Line is best fit. Note one trial where the motor behavior was highly delayed as was the timing on the digit electrode.

We next looked for temporal relationships with the onset of motor behavior. To do so, for each cluster, we compared the timing of nLFP occurrence at each electrode in each interval to the timing of behavior, which had an onset variability of 410±152 ms after the cue (Figure 6A). For this analysis we chose only those electrodes where sufficient data was available – i.e those where an nLFP occurred in a particular interval in at least 30% of the trials, and calculated the correlation coefficient between the timing of nLFP peak and the timing of the start of the behavior. In spite of the hand electrodes being significantly more active in the anticipation interval (Figure 4), nLFPs on none of the electrodes on any of the 9 clusters were correlated with R>0.5 with the timing of behavior. This result was expected since onset of the visual cue had a random variability of two seconds. In stark contrast, nLFPs occurring on electrodes 27 and 28 (digit electrodes, marked in circles in Figure 6A) in cluster RH1 (right fist) after onset of the cue (i.e. during the RT-ON) had a large positive correlation (R≥0.8) with the reaction time. Since an nLFP on one electrode was typically associated with cascades of activity in other electrodes within 25 ms (Figure 3D), we questioned whether the cascades were able to predict the reaction time more accurately. The mean cascade timing had a reasonable positive correlation with both RT-ON (R = 0.49) and RT-OFF (R = 0.74), however it was less accurate as compared to electrodes 27 and 28 individually (R = 0.80, 0.95, Table S8, Figures 6B and 6C). Correlations to nLFP timings in the response period were also positively skewed at all electrodes in RH1, likely representing rapid propagation of activity from the digit sites in the previous interval. However, it is significant that of 2124 calculated correlations (59 electrodes×4 intervals×9 clusters) between the nLFP timing and reaction time, only the timing on the digit sites after onset of the right fist cue were as strongly correlated (i.e. R≥0.8) demonstrating the temporal significance of the hand electrodes.

We also looked for evidence of a relationship between the number of nLFPs in the trial-associated coherence potentials and the strength of the EMG (defined as the area under the EMG curve). However, we found that this was not the case and number of nLFPs (summed in each interval separately) was unrelated to the EMG strength (mean correlation ± SD, −0.116±0.195).

### Fine scale distinction in temporal structure of behavior encoding Coherence Potential

**Figure 7:**
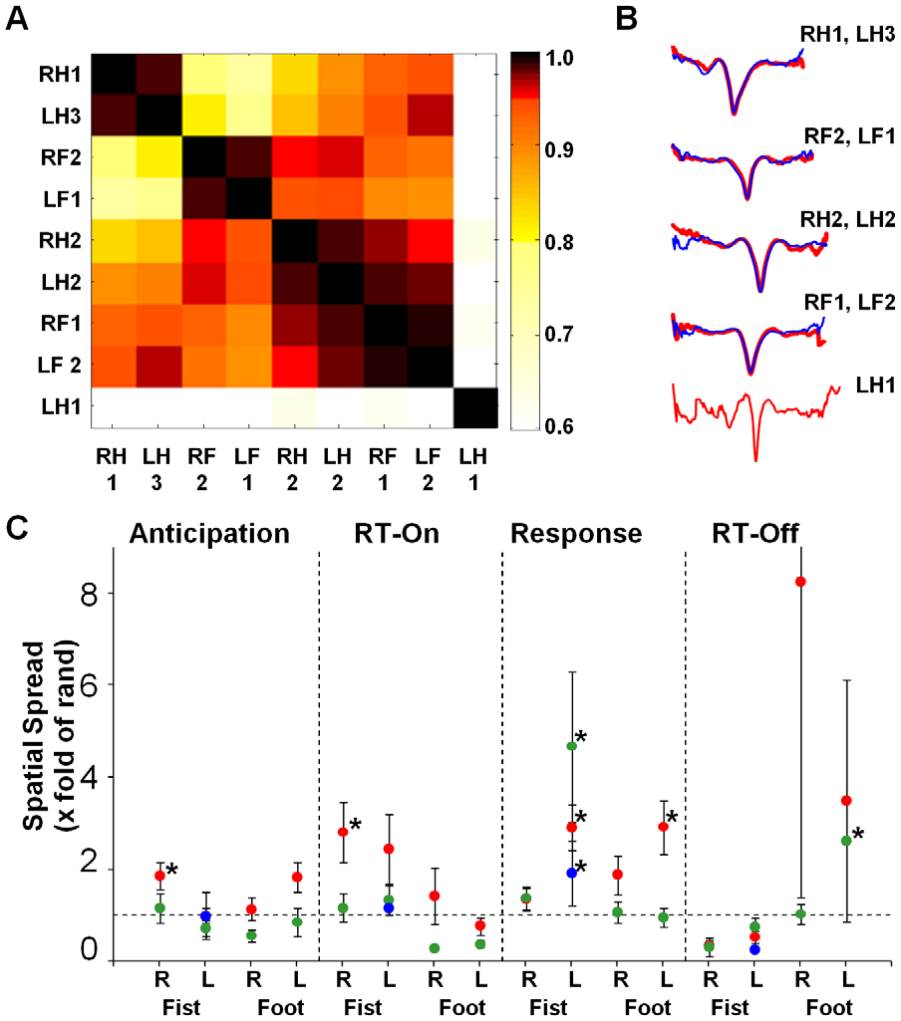
Fine Scale Temporal Structure of Coherence Potentials. (**A**) Correlations of the average nLFP waveform of each of nine clusters (9×9 matrix). Clusters RH1 (right fist clenching) and LH3 (left fist clenching) are highly similar as are clusters RF2 (right foot dorsiflexion) and LF1 (left foot dorsiflexion) but more distinct from one another. Clusters RH2, LH2, RF1 and LF2 are more similar to eachother. LH1 is distinct from the others. (**B**) Average waveforms compared in A. (**C**) Behaviorally relevant (i.e. trial associated) coherence potential waveforms have greater spatial spread than non-relevant waveforms. Spread within 10 ms after occurrence of an nLFP on electrodes 27 and 28 within each trial-spanning cluster normalized by the spread of other coherence potentials in the same interval that were not trial-spanning. The waveform of cluster RH1 (right fist clenching) had significantly greater spread in the anticipation and RT-ON intervals while clusters in the left fist clenching and left foot dorsiflexion had significantly greater spread in the response interval.

Our results show that it is only the occurrence of a particular coherence potential on the digit sites that predict the onset of fist clenching behavior and not any coherence potential. This suggests that the temporal structure of that particular coherence potential carries information or ‘message’ specific to the behavior. We thus compared the average nLFP of the coherence potentials (i.e. the nine clusters) across behaviors (Figure 7A) to see how similar or different their waveforms were. In all cases the tasks involved motor behaviors of varying similarity. We therefore expected to see gross similarities but fine scale differences (Figure 7B). Indeed while we found high similarity among the trial-spanning clusters that was greater than the similarity of comparisons of random nLFPs (>0.6 compared to 0.4; all clusters except cluster LH1), fine scale differences were evident. One of the clusters from right fist clenching (RH1) and one from left fist clenching (LH3) were highly similar (R>0.95) as were one of the clusters from left foot dorsiflexion (LF1) and one from right foot dorsiflexion (RF2). In contrast the two fist clusters were much more different from the two foot clusters (R<0.8) suggesting that they contained information about the particular body part to be moved. The other clusters (one from each behavior) were different from these two but more similar to one another suggesting that they convey non-specific information about the task (for e.g. initiating muscle movement). These findings suggest that subtle differences in the nature of movement were captured even at the LFP level.

We also asked if there might be fine scale differences in the message across intervals since the ‘message’ in anticipation (anticipating fist movement) would be different from the message after the onset of the cue (initiating fist movement). However, increasing clustering stringency did not result in a temporal separation of the cluster into interval specific groups (data not shown).

As another test of the information relevance of the nLFP structure we asked: when the LFP pattern encoding right fist clenching (RH1) occurred on the digit sites, was it more likely to spread further than other nLFP patterns of similar amplitude that were not relevant to the behavior. We thus compared the number of electrodes that mirrored the nLFP pattern within 10 ms following the occurrence on electrodes 27 and 28 (Figure 7C). Indeed we found that the coherence potential pattern relevant to the behavior (i.e. cluster RH1) engaged more electrodes within 10 ms than the average of all other coherence potentials that occurred on those electrode in both the anticipation and RT-ON intervals (1.8 fold and 2.8 fold of all other coherence potentials respectively, p<0.05 both cases). In addition, the behaviorally linked coherence potentials engaged more electrodes within 10 ms after occurrence on electrodes 27 and 28 in the left fist clenching behavior (LH1,LH2 and LH3, 2.9, 4.67 and 1.9 fold respectively, p<0.05 all cases) and left foot dorsiflexion during the motor response period (LF1, 2.92 fold, p<0.05).

Taken together our findings show that coherence potentials preferentially initiate at ‘expert sites’ positioned to initiate behavior and suggest that the extent of spread of message from expert sites are informed by the specific content of the message.

## Discussion

Coherence potentials are large amplitude negative periods in the LFP (nLFPs) with complex temporal structure that spread rapidly to other sites without distortion [35]. This phenomenon was previously discovered in spontaneous activity in monkeys and rat cultures and can be thought of as a network level action potential. However, their relationship to behavior was unknown. Here we have analyzed the relationship between coherence potentials and human motor behavior using electrocorticograph (ECoG) recordings from arrays of subdural electrodes coupled with EMG recordings of muscle movement in a human patient. We identified coherence potentials that consistently occurred across successive trials of a simple task where the subject mimicked a motor behavior shown in a visual cue. In these trial linked coherence potentials, we found that ‘expert sites’, i.e. those that resulted in a similar motor behavior on direct stimulation, were largely responsible for initiating the coherence potential. Furthermore, the timing of the coherence potential on the expert sites after onset of the cue predicted the timing of onset of the muscle movement. However, the spatial spread of the coherence potential was highly variable and most sites participated in just a small fraction of trials. The relationship was surprising but raises new hypothesis that may reconcile the disparate findings of gross localized function, fine scale response heterogeneity and distributed aspects of memory.

### Coherence Potentials across regions in human ECoG

In this study we have shown that Coherence Potentials are present in humans during a simple motor behavior task extending its presence to the behavioral paradigm. Of significance is that the original discovery of coherence potentials was made using electrodes that were typically 30 µm in diameter and restricted to a single region of the cortex in arrays spanning not more than 64 mm^2^ [35]. In these human ECoG recordings, the electrodes were 4 mm in diameter representing a ∼5900 fold decrease in spatial resolution. Furthermore the array spanned a total area of 44 cm^2^, an area almost 70 times as large extending across multiple functional regions from sensorimotor to language related frontal regions of the cortex. However despite these differences in spatial resolution, the primary result remained the same: As amplitude of the aggregate nLFP increased, the probability of finding the same temporal pattern at other electrodes increased in a non-linear fashion with similar time scales (Figure 2A) and extended across various combinations of all electrodes (Figures 4 and 5). This suggests a highly fractal or scale invariant nature to the phenomenon that is in line with earlier demonstrations of scale free dynamics in the cortex in both the spatial, temporal and amplitude domains [36], [37].

The consistency of these findings with the phenomenology in neuronal cultures and monkey cortex [35], [38] suggest that this is a generalizable result despite having been obtained from a single patient undergoing treatment for epilepsy. We further note that the results were obtained when the patient was normal functioning and that spectral properties of the signals reported in this dataset were consistent across multiple patients [6], [30], [34]

### Coherence Potentials Encode Behavior

The simplest hypothesis to describe a relationship between coherence potentials and behavior is that a particular coherence potential, i.e. a large amplitude nLFP with a distinct temporal structure, encodes information about the movement, and is consistently initiated at the sites responsible for hand movement. Indeed we found only a handful of coherence potentials that consistently spanned all trials occurring in rapid cascades that were punctuated by pauses that were time locked to the end of the behavior. Furthermore, we found that one particular coherence potential, arising during the right fist clenching behavior (RH1), was far more likely to engage the electrode regions that resulted in right hand movement on stimulation, during the anticipation of the cue and the reaction time after onset of the cue (RT-ON). Right hand region electrodes participated in the coherence potential in 78% of the trials during the anticipation period and in 52% of the trials during RT-ON compared to 50±12% and 31±10% trials (mean ± SD) for all other regions, and tended to lead in time during both periods. This was not the case for the coherence potentials during ipsilateral hand movement or either ipsi- or contralateral foot dorsiflexion where right hand electrodes were not distinct in their participation. Unfortunately the electrode array was specific to only one hemisphere and did not cover areas resulting in foot movement or sensation on stimulation. However, consistent with this, no dominant sites emerged during the other tasks. The occurrence of one particular coherence potential (RH1) on the right hand electrodes during the anticipation and RT-ON periods, and converse absence of participation during the response periods, was also highly significant relative to the overall distribution of all coherence potentials (i.e. large amplitude nLFPs; p<10^−3^) when coherence potential identities were shuffled.

The two electrodes (#27,28) that specifically resulted in right digit movement on stimulation also tended to lead immediately after the cue onset in this same coherence potential during the right fist clenching task. Furthermore, the timing at which this coherence potential occurred on these electrodes was highly correlated to the subsequent timing of motor behavior. Correlations between the duration from cue onset to coherence potential occurrence on these electrodes and duration from coherence potential occurrence to the onset of motor behavior were ≥0.8, far greater than any other participating electrodes. It is highly significant to note that the digits are the first to move during fist clenching.

These results thus draw a strong relationship between a particular coherence potential and behavior, indicating that the behavior is driven largely by the timing of when a particular coherence potential arises at ‘expert’ sites, thereby establishing both spatial and temporal significance of its occurrence. However, these ‘expert sites’ participated in only 78% and 52% of the trials in the anticipation and RT-ON intervals of the right fist task respectively, even though the behavior occurred consistently in all trials. One explanation for this inconsistency is that our electrode array did not cover the hand areas completely, (i.e. hand, wrist and finger areas), due to the wide electrode spacing and the surface position. In addition, previous studies have shown that large areas in the pre-central motor cortex including Brodmann areas 4 are known to play roles in finger movement based motor tasks [40]. These may serve as alternate expert sites that were outside of the fronto-parietal coverage of our electrode array. Such trial–to-trial variability may arise because different neurons or sets of neurons have different levels of priming to the particular behavior based on past history of activity. Certain ‘expert sites’ would therefore drive it with greater consistency than others because they are faster to respond to the stimulus. Variability in the neuronal groups leading and participating from trial-to-trial may actually play a critical role in establishing the trade-off between total information and redundancy between neurons in population codes [14].

### Information Content of Coherence Potentials

Synchrony among neurons and phase synchrony of oscillations, particularly in the gamma band, arise frequently during behavioural tasks and have been proposed to play a role in carrying behaviourally relevant information [41], [42]. Coherence potentials are highly structured periods of synchrony with specific phase relationships among many frequencies and therefore have far greater information depth than spectrally separated oscillations. The waveform or temporal structure of Coherence Potentials (i.e. of any given nLFP within a coherence potential) has been shown to reflect a synchronized temporal firing pattern of the neurons in the local field [37]. At the single neuron level, precise temporal firing sequences have been found in monkeys that are of the order of hundreds of ms [43], comparable to the average duration of nLFPs within a coherence potential. These sequences clustered in time during certain behaviour segments, suggesting a behavioural association and providing further evidence that the nLFP waveform reflects a propagating temporal code with many degrees of freedom.

Furthermore, a neuronal model based on simple parameters of spike timing dependent plasticity (STDP) and conduction delays produced large numbers of precise temporal patterns that occurred as polychronous events – i.e. repeating across different and sometimes overlapping groups of neurons in the network with brief time delays [44]. Moreover, particular persistent spike patterns were produced in response to stimuli indicating that they encode memory of the stimulus. Coherence Potentials, which are polychronous cascades could similarly emerge based on such self-organizing principles and could also be thought of as short term memories of the stimulus or task.

Relative to spike information, the aggregation and filtering of the signal across such a large spatial area in the LFP could easily obscure fine scale differences in the temporal code. Furthermore, the correlation measure might not be sufficiently powerful for small distinctions. To illustrate this point, take the sentences ‘Initiating fist movement’ and ‘Initiating foot movement’; if each letter translated to an amplitude value in the LFP, the corresponding correlation of the resulting time series would be very high and the small difference could easily be lost by spatial by filtering or obscured by noise. Nonetheless, in substantiation of a hypothesis that coherence potentials carry complex information, we were able to see fine scale distinctions in the temporal structure of the trial-spanning coherence potentials that grouped in a behaviourally relevant way (Figure 7A). Coherence potentials that were specific to right and left fist clenching were highly similar as were coherence potentials specific to right and left foot dorsiflexion. These two groups, however, were more distinct from one another. Such fine scale distinction has not been previously observed using spectral approaches. Earlier studies by Crone et. al. (using the same recording data) have shown event related synchronization and de-synchronization (ERS/ERD) of frequency components of the ECoG. They found an increase in the alpha (8–13 Hz) and beta (15–25 Hz) in the early and late phases of motor response, but not somatotopically specific to the action. However, they found a parallel increase in sustained gamma (30–45 Hz) activity and a transient increase in high frequency gamma activity (50–100 Hz) that were somatotopically specific [6], [30], [45].

### Spreading Coherence Potentials as ‘Knowledge-Sharing’: A Hypothesis

Synchrony on millisecond time scales has been proposed to play a role in binding of multimodal information [41]. Indeed coherence potentials represent a highly structured, rapid and precise synchrony where disparate regions of the cortex mirror one another in transient ‘agreement’. However, the spread of coherence potentials does not lend itself to a synchrony based theory of binding. First, binding or integration of multiple sources of information would involve processing on the time scales of propagation. However, coherence potentials, by definition involve the preservation of the message rather than further processing. Second, while there was a clear dominance of the right hand region in initiating a particular coherence potential during the anticipation and planning of the right fist clenching behavior, the overall number of sites participating in a cascade and the spatial spread varied widely across trials in each interval and bore no obvious relationship to the strength of the EMG response. Why then should the Coherence Potential spread?

Taking into consideration these various aspects, we propose a new framework for considering their purpose and brain function in general. We suggest that cross-modal binding may occur largely in sub-threshold activity (i.e. sub-threshold to the coherence potential definition and thus not in the context of widespread synchrony) in small ‘conversations’ among neurons. Expert sites, i.e. those sites which have greatest access to the most information about the task in question, may then form key messages that garner enough local support to spread across the cortex. We thus suggest that Coherence Potentials represent the spreading of information to ‘non-expert’ cortical areas, thereby building knowledge redundancy in the cortex that while not necessarily relevant for the short term, would confer longer term benefits such as in the event of damage or when greater resources are required for a larger task. While the formulation of expert messages would be difficult to parse out from the aggregate activity, their subsequent spread as coherence potentials allow easy identification, perhaps representing a view into the brain's ‘zeitgeist’. The degree of spread may also have a relationship to conscious awareness of the task and is a question for future investigation. Such a scenario has been alluded to in the Global Workspace Theory proposed by Bernard Baars [46]. In the experiment here, however, the tasks were extremely simple and repetitive and therefore would not require much focused awareness.

This framework has parallels to the functioning of society where knowledge is created at expert locations based on local and long range interactions and then disseminated across the world such that it may eventually become ‘common knowledge’ that everyone can use. Such ‘common knowledge’ in the cortex could explain the findings of Lashley and Hebb in the early and mid 20^th^ century that led to the theory of equipotentiality amidst increasing evidence of localized responses to stimulation [26]. Lashley found that the loss of memory in rats trained in various tasks depended largely on how much cortex was removed rather than the specific location that was removed, findings then extended by Hebb in the context of lesions in humans. However, lesions of specific regions prior to learning of a task resulted in an inability to learn. In this framework of origination and dissemination from ‘expert’ sites leading to common knowledge, it is also possible to reconcile gross functional localization with high variability of responses in individual neurons, heterogeneity in the response profiles of neighboring neurons and the theory of distributed knowledge or memory.

## Materials and Methods

### Ethics Statement

The protocol is approved by Joint Committee on Clinical Investigation of The Johns Hopkins Medical Institutions in compliance with standards of Declaration of Helsinki. The experiments were done with informed written consent from all the participants of the study. Several papers have been published using the same dataset [6], [30].

### Electrode Implantation

The recordings were done in a 31 year old right handed male subject admitted for intractable epilepsy treatment with his informed consent. An array of subdural electrodes was implanted on the surface in the left fronto-parietal region. The subdural grid was 1.5 mm thick silastic sheet embedded with Platinum-Iridium electrodes (4 mm outer diameter, 2.3 mm exposed diameter). The electrodes were equally spaced with a centre to centre distance of 1 cm. The electrodes remained implanted for a period of 4–13 days during which the subject participated in these experiments when he was awake. The seizure focus was in left frontal region and the lesioned region was in the left inferior frontal encephalomalacia.

### Functional Mapping

Sensorimotor localization of brain function was mapped with cortical electrical stimulation [7], [30]. Current was passed between pairs of adjacent electrodes. The current pattern was 1–5 s trains at 50 Hz, 0.3 ms duration alternating polarity square wave pulses. The stimulus intensity was varied between 1–15 mA in increments of 0.5 mA. Movement was observed visually and the patient reported sensations. Language function was assessed with specific tasks during the stimulation.

### Behavioral Task

The task required the subject to make sustained voluntary muscle contractions in response to a visual cue on the screen. The visual cues were black and white drawings depicting motor actions – fist clenching of one arm and dorsiflexion of one foot. We collected data for 4 tasks – right arm, left arm, right foot and left foot. In every session only one of the 4 tasks was repeated for 50 trials. The visual cue was shown for 3 seconds and the interval between consecutive trials was randomly selected between 3 and 5 seconds. In the absence of a visual cue the subject was asked to fix his gaze at a black dot at the centre of the screen.

### Extraction of Coherence Potentials

The recorded signals were amplified by 5000X and filtered using a band-pass filter at 1–100 Hz. The resulting local field potentials (LFPs) in the ECoG recordings from 59 electrode channels were scanned for negative periods or nLFPs whose peaks exceeded a certain amplitude criteria. Time-matched and time-shifted correlations (Figure 2A) were done using 300 nLFPs per trigger electrode selected at random for each amplitude value (in multiples of SD from the mean) [35].

For further analysis we defined the threshold for nLFP detection as 2 standard deviations from the mean for each electrode channel. For each task we extracted between 9586–11050 suprathreshold nLFPs from 59 electrodes across 50 trials.

Each nLFP was then compared to all other nLFPs by calculating the correlation coefficient after peak alignment to produce a N×N correlation matrix, where N is the number of supra-threshold nLFPs (Figure 2B). This provided us a measure of temporal similarity of the waveform independent of their amplitudes. Since the comparisons had to be periods of equal duration we used the maximum period length before and after the peaks for the nLFP pair being compared. The nLFPs were then clustered based on correlation values using the dendrogram function in MATLAB (minimum all-to-all). We varied the correlation value of clustering (stringency) between 0.05 to 1 (1 being maximum correlation) for the dendrogram function. At a clustering stringency of 0.7 we scanned all the clusters and found clusters that had nLFPs in all 50 trials. These clusters were defined as trial-spanning clusters. Given the long gaps between trials relative to other trial segments and therefore the large number of nLFPs in this segment, the clustering was carried out by excluding the first few seconds after a trial ended, and starting instead 1 second before the onset of the cue in each trial. These 9 resulting trial spanning clusters were used for all subsequent analysis except in the determination of inter-trial intervals where clustering using all nLFPs in the inter-trial period was used. This additional inclusion resulted in similar clusters. However, in three out of four behaviors this resulted in splintering into an additional cluster at the 0.7 cut-off.

### Analysis of Coherence Potentials

The quality and separation of clusters were further assessed using silhouette coefficients. Silhouette Coefficient,
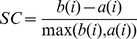
(1)where *a(i)* is the average distance to the points in the cluster and *b(i)* is the minimum average distance to the points in another cluster. The value of SC for any nLFP can vary between −1 and 1. SC value closer to 1 means the nLFP has been accurately classified.

To analyze the spatiotemporal distribution of nLFPs in each cluster we divided each trial into four intervals based on information about the motor behavior derived from the EMG: Anticipation: from muscle relaxation at end of the last trial to onset of cue of the next trial, Reaction Time – ON (RT-ON): the time from onset of the cue to the time of onset of muscle contraction or motor behavior, Response time: – the duration of the motor behavior and Reaction Time – OFF (RT-OFF): the time from when the cue is turned off to muscle relaxation. The duration of motor behavior (fist clenching/foot dorsiflexion) was defined by the start and end points of the EMG. These were defined as the time points when excursion of the rate of the EMG (voltage change) was 2 standard deviations away from the mean voltage rate.

The statistical significance of the spatiotemporal maps (Figure 5) was estimated using a boot-strapping analysis (Figure S6). The timing of all nLFPs>2 SD for a given behavioral task were shuffled. This gave us clusters with same numbers of nLFPs but now randomly distributed at different electrodes and time periods. We generated 5000 such shuffled sets each with a different spatiotemporal map. The p-values were then estimated as the probability of a given electrode having significantly higher or lower trial participation or temporal rank than the shuffled sets. As a further test of spatiotemporal specificity of particular electrodes during the task the spatiotemporal analysis was repeated after removing nLFPs with a SC<0.5 (Eqn. 1) from the nine trial-spanning clusters to reveal the core of the clusters (Figure S5).

Raster plots of the nLFP timing (Figure 4C) showed that sometimes nLFPs tend to occur in quick succession (<10 ms) across a large number of electrodes. To determine whether this cascade was related to behavior we divided the time axis into 25 ms bins and grouped the nLFPs arising in each bin cluster-wise. We then calculated the average time with the bin which had the largest number of nLFPs and correlated it with the duration of the reaction times. This was done independently for each of the four intervals. We found positive correlations between the cascades occurring during RT-ON with duration of RT-ON in all 9 clusters (Table S8). We also found positive correlations between the cascades occurring during RT-OFF and duration of RT-OFF in all 9 clusters. All analyses were done using MATLAB 7.0.

## Supporting Information

Figure S1 **Trial Spanning Clusters.** (**A, C, E**) Cluster dispersion across trials for different correlation criteria for clustering in the left fist clenching, right and left foot dorsiflexion tasks respectively. At a low correlation criterion (light gray) all nLFPs collapse into a few clusters and thus span all trials (trial-spanning clusters). At higher correlation criteria, clusters get splintered and most clusters span only a few trials. However, a few clusters span all trials. R = 0.7 was chosen for further analysis. (**B, D, F**) Splintering of trial-spanning clusters with increasing correlation criteria for clustering in the left fist clenching, right and left foot dorsiflexion tasks respectively. The lines connect the parent cluster from which smaller clusters have separated. Only trial-spanning clusters are shown in the figure. We find three, two and two trial spanning clusters at R = 0.7 in each of the three tasks.
(PDF)

Figure S2 **Coherence Potential Cascades Temporally Associated to Trials.** (**A**) Distributions of the intervals between the last nLFP of one trial and the first nLFP of the next trial of all nine trial spanning clusters (dotted line) were significantly longer than the inter-nLFP intervals within the trials (solid line) indicating clusters are fast trial associated cascades punctuated by longer pauses at the end of the trial. (**B**) Cumulative histogram of panel (A).
(PDF)

Figure S3 **Cluster Size Distribution.** (**A**) Distribution of cluster sizes in all four behavioral tasks indicates a power law relationship, a signature of neuronal avalanches. Cluster size is measured as the number of nLFPs (>2SD) in the cluster.
(PDF)

Figure S4 **Coherence Potentials Trial Spanning Clusters.** (**A**) Distribution of number of nLFPs in each trial shown for the 9 trial spanning clusters. Each panel contains the trial spanning clusters for a particular behavioral task. The distribution spreads across a large range is almost uniform for the large clusters.
(PDF)

Figure S5 **Spanning Clusters show clear interval associated spatiotemporal patterns after enrichment using Silhouette Coefficients.** (**A**) Spatiotemporal patterns of nLFPs in two out of nine trial-spanning clusters for each interval. These clusters have been enriched by removing nLFPs with silhouette coefficients <0.5 demonstrating that in the right fist clenching task the ‘expert sites’ (hand electrodes) remain at the centre or core of the cluster. (Circle size: trial participation; Color intensity: mean rank of temporal occurrence early (black) to late (white)).
(PDF)

Figure S6 **Digit electrodes lead cascades in right fist clenching task.** (**A**) Distribution of p-values for the 59 electrodes (refer Figure 5) shows that only a few electrodes have the nLFPs occurring more often and earlier in the trial in only cluster RH1. (**B–C**) Comparison between clusters with electrode color-coded based on their functional map (Figure 1B) shows higher mean rank (comes earlier) for the hand electrodes (marked in red) belonging to cluster RH1.
(PDF)

Table S1 Table shows the mean and standard deviation of the distribution of the trial-spanning clusters. Distance was calculated as (1-correlation between the nLFPs).
(DOC)

Table S2 Table shows the mean SC and percentage of SCs greater than zero for the nLFPs belonging to the nine trial spanning clusters. SC greater than zero, indicates accurate clustering.
(DOC)

Table S3 Table shows the mean ± standard deviation of the interval durations over 50 trials for each of the four behavioral tasks.
(DOC)

Table S4 Table shows the percentage (mean ± standard deviation) of electrodes participation in a trial averaged over 50 trials for each of the four behavioral tasks.
(DOC)

Table S5 Table is a list of the electrodes which have significantly higher (p<0.005) occurrences of 1^st^ nLFP belonging to the trial-spanning clusters across 50 trials. Significance was calculated using boot-strapping across 5000 iterations.
(DOC)

Table S6 Table is a list of the electrodes where the nLFP arise significantly more/less often (p<0.005, Boot-strapping analysis 5000 iterations) across 50 trials (also see Figure 5).
(DOC)

Table S7 Table is a list of the electrodes where the nLFP arise significantly earlier/later (p<0.005, Boot-strapping analysis 5000 iterations) as calculated by the mean rank (Methods) (also see Figure 5).
(DOC)

Table S8 Table shows the correlation and linear regression fit coefficient (R^2^) values between the mean LFP timing of the largest cascade during RT-ON (left) and duration of RT-ON. On the right are the same values for RT-OFF.
(DOC)
