# Supplementary figures and images for: Coherence Potentials Encode Simple Human Sensorimotor Behavior
Source: PLoS One. 2012 Feb 3;7(2):e30514. doi: 10.1371/journal.pone.0030514 (PMC3272042; doi:10.1371/journal.pone.0030514)

**Figure S1**

**Left Fist**

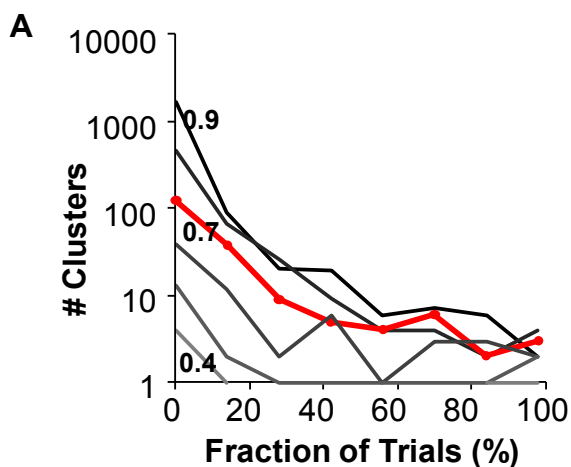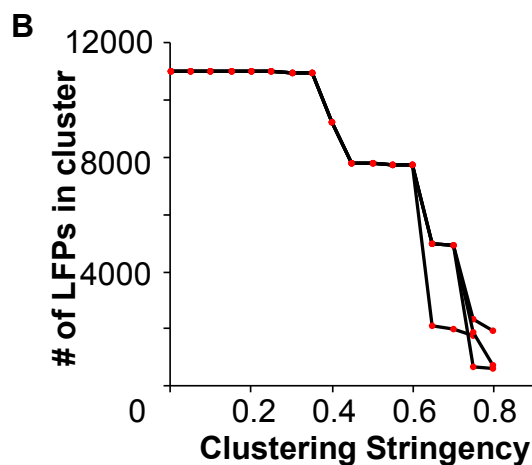

**Right Foot**

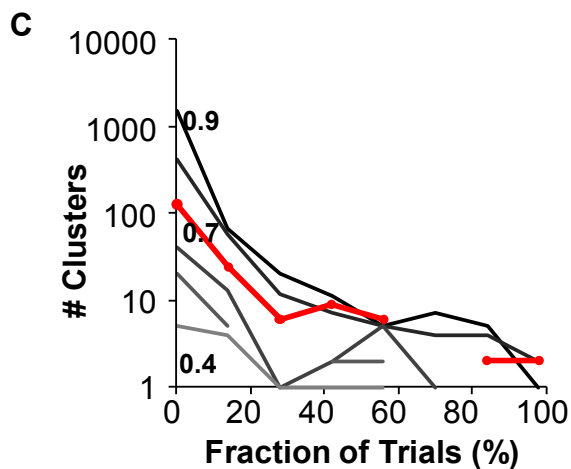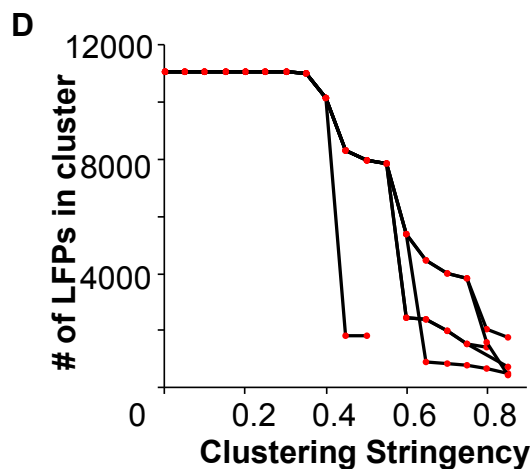

**Left Foot**

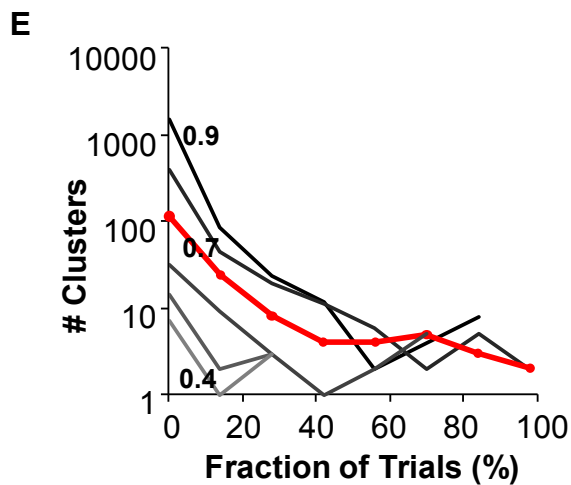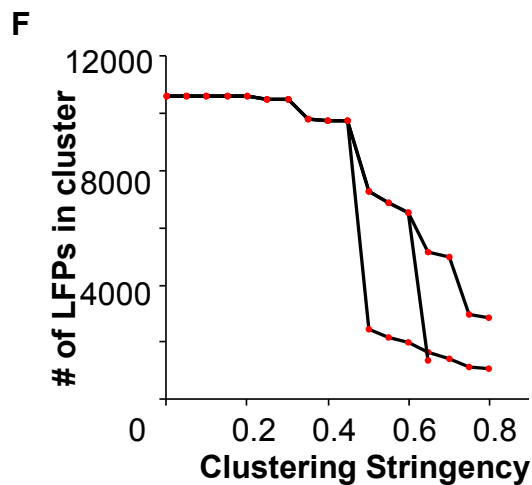

Supplement: Figure S1 — Trial Spanning Clusters. (A, C, E) Cluster dispersion across trials for different correlation criteria for clustering in the left fist clenching, right and left foot dorsiflexion tasks respectively. At a low correlation criterion (light gray) all nLFPs collapse into a few clusters and thus span all trials (trial-spanning clusters). At higher correlation criteria, clusters get splintered and most clusters span only a few trials. However, a few clusters span all trials. R = 0.7 was chosen for further analysis. (B, D, F) Splintering of trial-spanning clusters with increasing correlation criteria for clustering in the left fist clenching, right and left foot dorsiflexion tasks respectively. The lines connect the parent cluster from which smaller clusters have separated. Only trial-spanning clusters are shown in the figure. We find three, two and two trial spanning clusters at R = 0.7 in each of the three tasks. (PDF) [file pone.0030514.s001.pdf]

Figure S2: Coherence Potential Cascades Temporally Associated to Trials

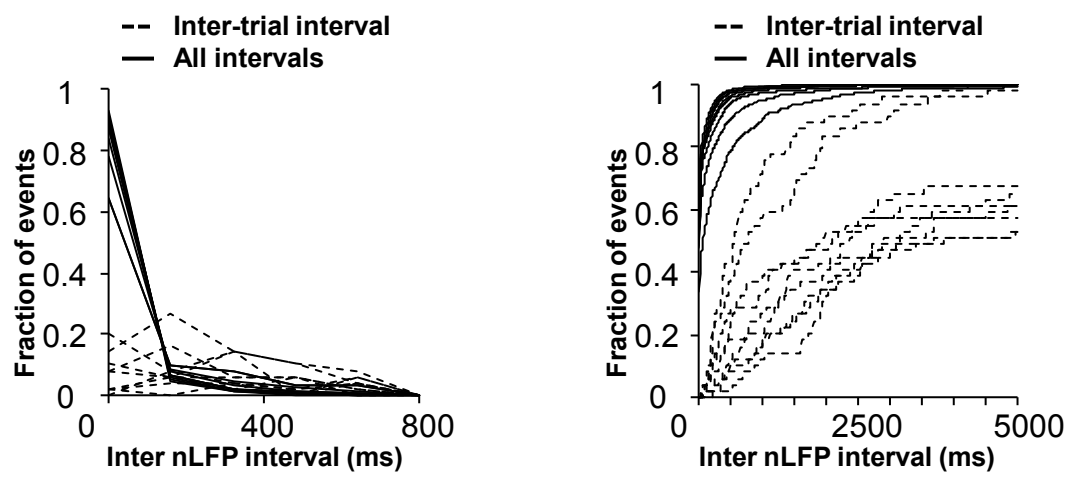

Supplement: Figure S2 — Coherence Potential Cascades Temporally Associated to Trials. (A) Distributions of the intervals between the last nLFP of one trial and the first nLFP of the next trial of all nine trial spanning clusters (dotted line) were significantly longer than the inter-nLFP intervals within the trials (solid line) indicating clusters are fast trial associated cascades punctuated by longer pauses at the end of the trial. (B) Cumulative histogram of panel (A). (PDF) [file pone.0030514.s002.pdf]

**Figure S3: Cluster size Distribution**

**A**

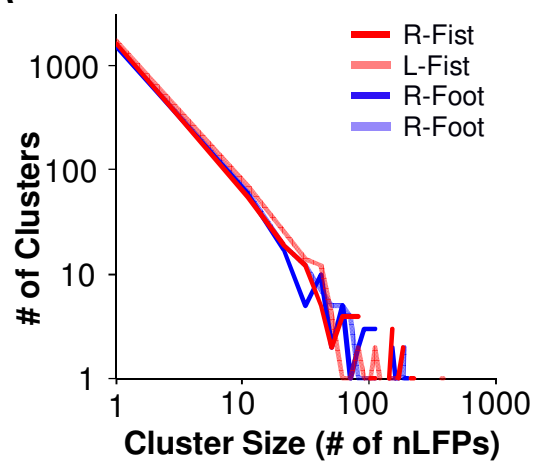

Supplement: Figure S3 — Cluster Size Distribution. (A) Distribution of cluster sizes in all four behavioral tasks indicates a power law relationship, a signature of neuronal avalanches. Cluster size is measured as the number of nLFPs (>2SD) in the cluster. (PDF) [file pone.0030514.s003.pdf]

**Figure S4: Coherence Potentials within trial-spanning clusters**

**A**

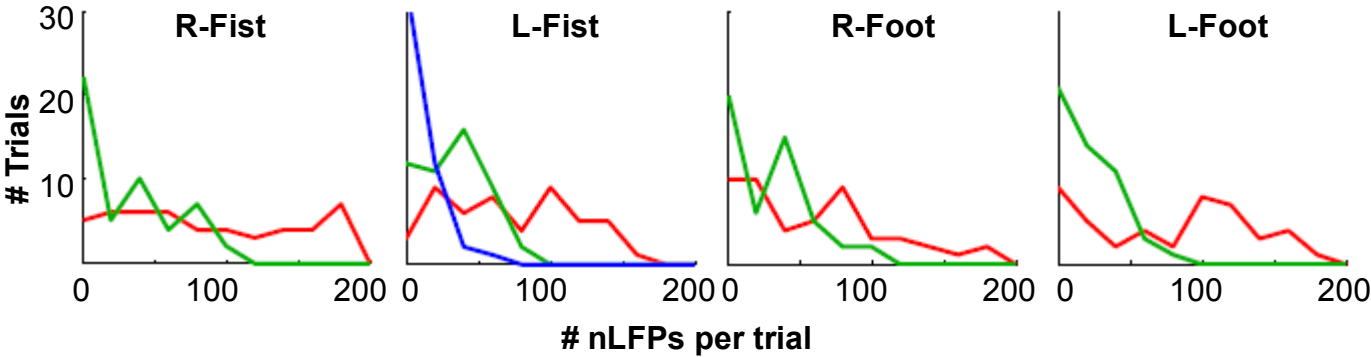

Supplement: Figure S4 — Coherence Potentials Trial Spanning Clusters. (A) Distribution of number of nLFPs in each trial shown for the 9 trial spanning clusters. Each panel contains the trial spanning clusters for a particular behavioral task. The distribution spreads across a large range is almost uniform for the large clusters. (PDF) [file pone.0030514.s004.pdf]

**Figure S6: Digit electrodes lead cascades in right fist clenching task**

**A**

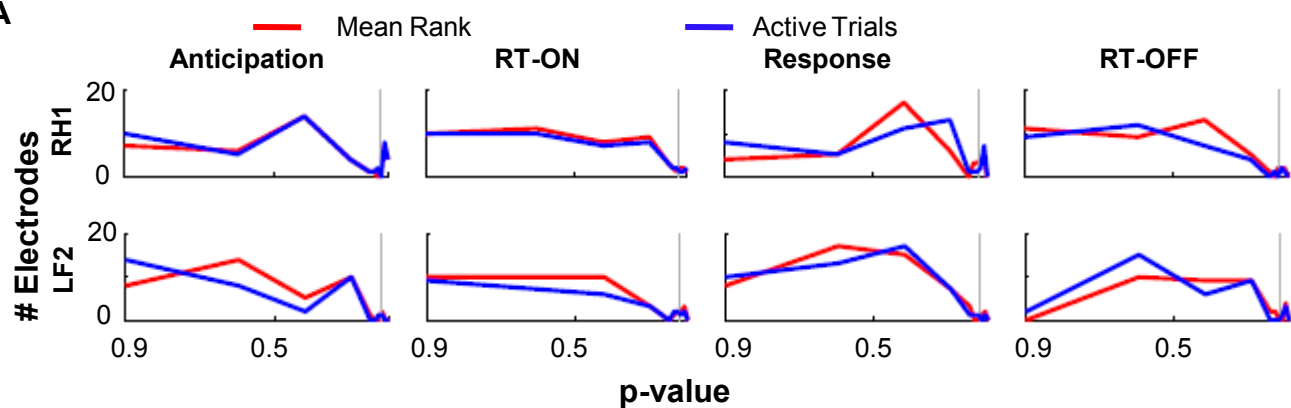

**B**

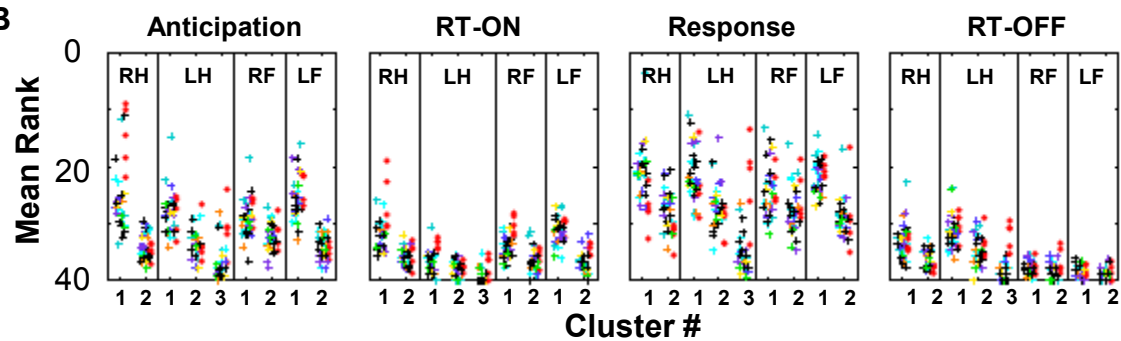

**C**

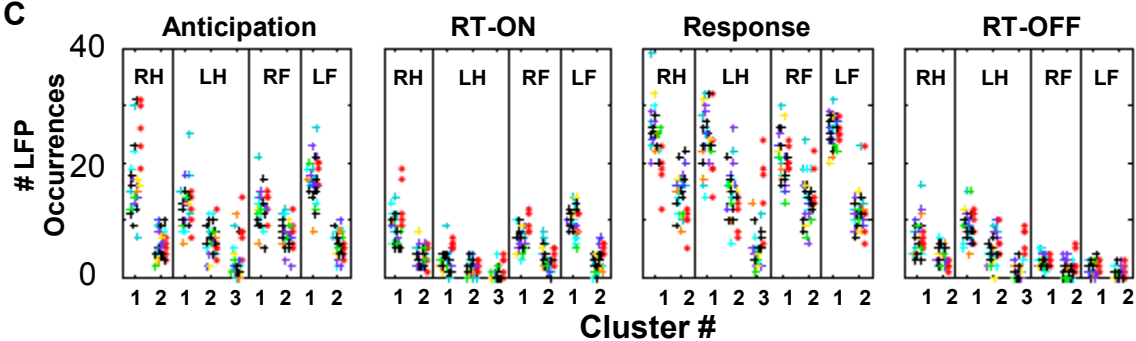

Supplement: Figure S6 — Digit electrodes lead cascades in right fist clenching task. (A) Distribution of p-values for the 59 electrodes (refer Figure 5) shows that only a few electrodes have the nLFPs occurring more often and earlier in the trial in only cluster RH1. (B–C) Comparison between clusters with electrode color-coded based on their functional map (Figure 1B) shows higher mean rank (comes earlier) for the hand electrodes (marked in red) belonging to cluster RH1. (PDF) [file pone.0030514.s006.pdf]
