# Supplementary material for: Coherence Potentials Encode Simple Human Sensorimotor Behavior
Source: PLoS One. 2012 Feb 3;7(2):e30514. doi: 10.1371/journal.pone.0030514 (PMC3272042; doi:10.1371/journal.pone.0030514)

Figure S5: Spanning Clusters show clear interval associated spatiotemporal patterns after enrichment using Silhouette Coefficients

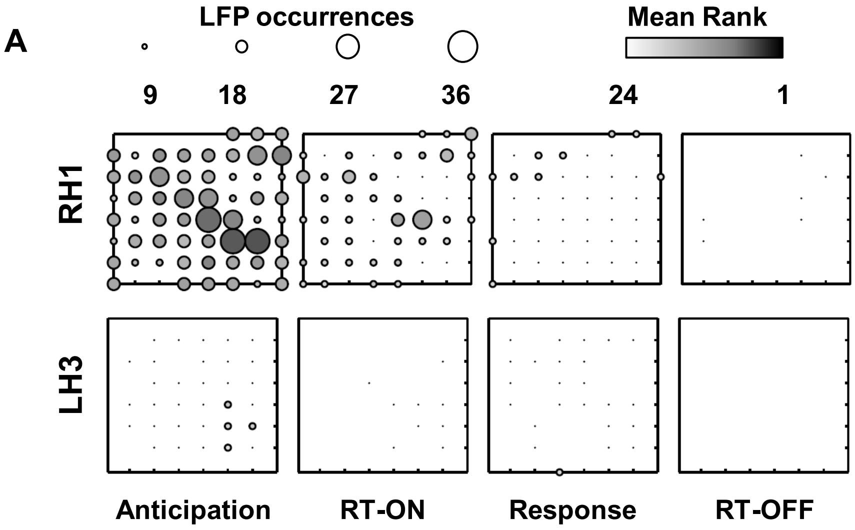

Supplement: Figure S5 — Spanning Clusters show clear interval associated spatiotemporal patterns after enrichment using Silhouette Coefficients. (A) Spatiotemporal patterns of nLFPs in two out of nine trial-spanning clusters for each interval. These clusters have been enriched by removing nLFPs with silhouette coefficients <0.5 demonstrating that in the right fist clenching task the ‘expert sites’ (hand electrodes) remain at the centre or core of the cluster. (Circle size: trial participation; Color intensity: mean rank of temporal occurrence early (black) to late (white)). (PDF) [file pone.0030514.s005.pdf]
