# Supplementary material for: Coherence Potentials Encode Simple Human Sensorimotor Behavior
Source: PLoS One. 2012 Feb 3;7(2):e30514. doi: 10.1371/journal.pone.0030514 (PMC3272042; doi:10.1371/journal.pone.0030514)
Supplement: Table S1 — Table shows the mean and standard deviation of the distribution of the trial-spanning clusters. Distance was calculated as (1-correlation between the nLFPs). (DOC) [file pone.0030514.s007.doc]

**TITLE: Coherence potentials encode human motor behavior**

**Supporting Table S1**

| **Cluster** | **Mean** *a(i)* | **SD** *a(i)* |
| --- | --- | --- |
| **RH1** | 0.2313 | 0.0815 |
| **RH2** | 0.2062 | 0.0621 |
| **LH1** | 0.1951 | 0.0619 |
| **LH2** | 0.2088 | 0.071 |
| **LH3** | 0.1502 | 0.072 |
| **RF1** | 0.1775 | 0.0609 |
| **RF2** | 0.2248 | 0.0783 |
| **LF1** | 0.2402 | 0.0809 |
| **LF2** | 0.1944 | 0.0673 |
