# Supplementary material for: Coherence Potentials Encode Simple Human Sensorimotor Behavior
Source: PLoS One. 2012 Feb 3;7(2):e30514. doi: 10.1371/journal.pone.0030514 (PMC3272042; doi:10.1371/journal.pone.0030514)
Supplement: Table S2 — Table shows the mean SC and percentage of SCs greater than zero for the nLFPs belonging to the nine trial spanning clusters. SC greater than zero, indicates accurate clustering. (DOC) [file pone.0030514.s008.doc]

**TITLE: Coherence potentials encode human motor behavior**

**Supporting Table S2**

| **Cluster** | **Mean SC** | **SC>0 (%)** |
| --- | --- | --- |
| **RH1** | 0.2876 | 70 |
| **RH2** | 0.2761 | 73 |
| **LH1** | 0.3574 | 86 |
| **LH2** | 0.423 | 90 |
| **LH3** | 0.4401 | 90 |
| **RF1** | 0.6346 | 99 |
| **RF2** | 0.4086 | 90 |
| **LF1** | 0.2836 | 71 |
| **LF2** | 0.285 | 72 |
