# Supplementary material for: Coherence Potentials Encode Simple Human Sensorimotor Behavior
Source: PLoS One. 2012 Feb 3;7(2):e30514. doi: 10.1371/journal.pone.0030514 (PMC3272042; doi:10.1371/journal.pone.0030514)
Supplement: Table S3 — Table shows the mean ± standard deviation of the interval durations over 50 trials for each of the four behavioral tasks. (DOC) [file pone.0030514.s009.doc]

**TITLE: Coherence potentials encode human motor behavior**

**Supporting Table S3**

| Duration (in ms) | **RT-ON** | **Response** | **RT-OFF** |
| --- | --- | --- | --- |
| **R-Fist** | 410 ± 152 | 2478 ± 475 | 377 ± 165 |
| **L-Fist** | 331 ± 157 | 2645 ± 156 | 434 ± 154 |
| **R-Foot** | 468 ± 119 | 2434 ± 476 | 189 ± 101 |
| **L-Foot** | 454 ± 171 | 2470 ± 394 | 192 ± 134 |
