# Supplementary material for: Coherence Potentials Encode Simple Human Sensorimotor Behavior
Source: PLoS One. 2012 Feb 3;7(2):e30514. doi: 10.1371/journal.pone.0030514 (PMC3272042; doi:10.1371/journal.pone.0030514)
Supplement: Table S4 — Table shows the percentage (mean ± standard deviation) of electrodes participation in a trial averaged over 50 trials for each of the four behavioral tasks. (DOC) [file pone.0030514.s010.doc]

**TITLE: Coherence potentials encode human motor behavior**

**Supporting Table S4**

| Cluster Name (%) | **Anticipation** | **RT-ON** | **Response** | **RT-OFF** |
| --- | --- | --- | --- | --- |
| **RH1** | 33.4 ± 10.2 | 17.3 ± 6.5 | 50.3 ± 10.0 | 13.8 ± 5.2 |
| **RH2** | 12.4 ± 4.9 | 7.6 ± 3.8 | 30.1 ± 7.4 | 7.3 ± 3.1 |
| **LH1** | 24.9 ± 7.8 | 6.6 ± 4.0 | 50.6 ± 10.8 | 17.7 ± 6.1 |
| **LH2** | 13.4 ± 5.2 | 4.6 ± 2.6 | 31.6 ± 8.7 | 10.1 ± 4.1 |
| **LH3** | 6.0 ± 5.6 | 1.3 ± 2.1 | 13.5 ± 9.1 | 3.5 ± 4.1 |
| **RF1** | 23.9 ± 6.6 | 13.5 ± 4.6 | 42.5 ± 9.4 | 5.3 ± 2.0 |
| **RF2** | 14.1 ± 5.3 | 6.9 ± 3.7 | 28.2 ± 7.7 | 3.0 ± 2.6 |
| **LF1** | 34.2 ± 7.6 | 19.7 ± 4.5 | 53.5 ± 6.0 | 3.9 ± 2.1 |
| **LF2** | 10.7 ± 4.4 | 5.6 ± 3.7 | 23.9 ± 6.9 | 1.8 ± 1.8 |
