# Supplementary material for: Coherence Potentials Encode Simple Human Sensorimotor Behavior
Source: PLoS One. 2012 Feb 3;7(2):e30514. doi: 10.1371/journal.pone.0030514 (PMC3272042; doi:10.1371/journal.pone.0030514)
Supplement: Table S5 — Table is a list of the electrodes which have significantly higher (p<0.005) occurrences of 1st nLFP belonging to the trial-spanning clusters across 50 trials. Significance was calculated using boot-strapping across 5000 iterations. (DOC) [file pone.0030514.s011.doc]

**TITLE: Coherence potentials encode human motor behavior**

**Supporting Tables**

| **1^st^ nLFP** | **Anticipation** | **RT-ON** | **Response** | **RT-OFF** |
| --- | --- | --- | --- | --- |
| **RH1** | 28 | 27 |  |  |
| **RH2** |  | 27 | 59 |  |
| **LH1** |  |  |  | 41 |
| **LH2** |  |  |  |  |
| **LH3** |  |  | 55 |  |
| **RF1** |  |  | 57 |  |
| **RF2** |  |  | 48 |  |
| **LF1** | 27 |  | 28 |  |
| **LF2** |  |  |  |  |
