# Supplementary material for: Coherence Potentials Encode Simple Human Sensorimotor Behavior
Source: PLoS One. 2012 Feb 3;7(2):e30514. doi: 10.1371/journal.pone.0030514 (PMC3272042; doi:10.1371/journal.pone.0030514)
Supplement: Table S6 — Table is a list of the electrodes where the nLFP arise significantly more/less often (p<0.005, Boot-strapping analysis 5000 iterations) across 50 trials (also see Figure 5). (DOC) [file pone.0030514.s012.doc]

**TITLE: Coherence potentials encode human motor behavior**

**Supporting Table S6**

| **nLFP occurs** | **Anticipation** | **RT-ON** | **Response** | **RT-OFF** |
| --- | --- | --- | --- | --- |
| **RH1** | 10,12,18,19,27,28,36, 37, 49 | 27, 28 | 19,27,28,32,34, 46, 47 | 53 |
| **RH2** |  |  | 3 |  |
| **LH1** |  | 4 | 46 |  |
| **LH2** | 23 | 17 | 31 |  |
| **LH3** |  |  |  |  |
| **RF1** |  | 28, 57 | 51 | 59 |
| **RF2** |  |  | 17 | 28 |
| **LF1** | 57 | 1 |  |  |
| **LF2** |  | 32 |  |  |
