# Supplementary material for: Coherence Potentials Encode Simple Human Sensorimotor Behavior
Source: PLoS One. 2012 Feb 3;7(2):e30514. doi: 10.1371/journal.pone.0030514 (PMC3272042; doi:10.1371/journal.pone.0030514)
Supplement: Table S7 — Table is a list of the electrodes where the nLFP arise significantly earlier/later (p<0.005, Boot-strapping analysis 5000 iterations) as calculated by the mean rank (Methods) (also see Figure 5). (DOC) [file pone.0030514.s013.doc]

**TITLE: Coherence potentials encode human motor behavior**

**Supporting Table S7**

| **Mean Rank** | **Anticipation** | **RT-ON** | **Response** | **RT-OFF** |
| --- | --- | --- | --- | --- |
| **RH1** | 10,18,19,27, 28,36, 37,49 | 27, 28 | 19, 27, 28, 47 | 26, 53 |
| **RH2** |  | 27 | 3, 48 |  |
| **LH1** |  | 4 | 1, 46, 48 | 5 |
| **LH2** | 16, 23 |  | 31 |  |
| **LH3** |  | 12 | 22 |  |
| **RF1** | 40 | 57 | 48, 57 | 59 |
| **RF2** |  | 16 |  | 20, 28 |
| **LF1** | 57 | 1 |  |  |
| **LF2** |  | 21 |  |  |
