# Supplementary material for: Coherence Potentials Encode Simple Human Sensorimotor Behavior
Source: PLoS One. 2012 Feb 3;7(2):e30514. doi: 10.1371/journal.pone.0030514 (PMC3272042; doi:10.1371/journal.pone.0030514)
Supplement: Table S8 — Table shows the correlation and linear regression fit coefficient (R2) values between the mean LFP timing of the largest cascade during RT-ON (left) and duration of RT-ON. On the right are the same values for RT-OFF. (DOC) [file pone.0030514.s014.doc]

**TITLE: Coherence potentials encode human motor behavior**

**Supporting Table S8**

| **RT-ON Cascade correlation** | | |
| --- | --- | --- |
| **Cluster #** | **R-square** | **Corr** |
| RH1 | 0.02 | 0.49 |
| RH2 | 0.71 | 0.84 |
| LH1 | 0.32 | 0.56 |
| LH2 | 0.36 | 0.6 |
| LH3 | 0.42 | 0.65 |
| RF1 | 0.33 | 0.58 |
| RF2 | 0.56 | 0.75 |
| LF1 | 0.35 | 0.59 |
| LF2 | 0.04 | 0.06 |

| **RT-OFF Cascade correlation** | | |
| --- | --- | --- |
| **Cluster #** | **R-square** | **Corr** |
| RH1 | 0.55 | 0.74 |
| RH2 | 0.16 | 0.4 |
| LH1 | 0.18 | 0.42 |
| LH2 | 0.39 | 0.63 |
| LH3 | 0.21 | 0.45 |
| RF1 | 0.39 | 0.63 |
| RF2 | 0.91 | 0.95 |
| LF1 | 0.91 | 0.95 |
| LF2 | 0.9 | 0.95 |
